# Torsional nystagmus recognition based on deep learning for vertigo diagnosis

**DOI:** 10.3389/fnins.2023.1160904

**Published:** 2023-06-09

**Authors:** Haibo Li, Zhifan Yang

**Affiliations:** College of Electronic and Electrical Engineering, Shanghai University of Engineering Science, Shanghai, China

**Keywords:** torsional nystagmus, deep learning, classification and identification, convolution network, benign paroxysmal positional vertigo

## Abstract

**Introduction:**

Detection of torsional nystagmus can help identify the canal of origin in benign paroxysmal positional vertigo (BPPV). Most currently available pupil trackers do not detect torsional nystagmus. In view of this, a new deep learning network model was designed for the determination of torsional nystagmus.

**Methods:**

The data set comes from the Eye, Ear, Nose and Throat (Eye&ENT) Hospital of Fudan University. In the process of data acquisition, the infrared videos were obtained from eye movement recorder. The dataset contains 24521 nystagmus videos. All torsion nystagmus videos were annotated by the ophthalmologist of the hospital. 80% of the data set was used to train the model, and 20% was used to test.

**Results:**

Experiments indicate that the designed method can effectively identify torsional nystagmus. Compared with other methods, it has high recognition accuracy. It can realize the automatic recognition of torsional nystagmus and provides support for the posterior and anterior canal BPPV diagnosis.

**Discussion:**

Our present work complements existing methods of 2D nystagmus analysis and could improve the diagnostic capabilities of VNG in multiple vestibular disorders. To automatically pick BPV requires detection of nystagmus in all 3 planes and identification of a paroxysm. This is the next research work to be carried out.

## 1. Introduction

The vestibular system informs us of three-dimensional (3D) head position in space. Vestibular asymmetry creates a hallucination of head movement and therefore generates a compensatory slow phase eye movement and a quick phase that returns the eye closer to its starting position. Nystagmus is an involuntary oscillating eye movement that accompanies vestibular disorders (Leigh and Zee, 2015). The involvement of a specific vestibular end organ can be identified by the nystagmus trajectory (Jiang et al., 2018). Nystagmus can be summarized into two types: pathological nystagmus and physiological nystagmus. A variety of diseases, such as BPPV, Meniere’s disease and vestibular neuritis, are all associated with pathological nystagmus (Newman et al., 2019). Pathological nystagmus arises from asymmetries in the peripheral or central vestibular system. Physiological nystagmus can be generated by rotational or thermal stimulation of the vestibular system (Henriksson, 1956; Cohen et al., 1977). BPPV is usually accompanied by nystagmus, which provoked by changes of the head position relative to gravity (Lim et al., 2019). To diagnose different types of BPPV, clinicians inspect the directional and velocity characteristics of positional nystagmus during provocative testing. Among them, for BPPV with the highest incidence rate in the posterior semicircular canal, the typical torsional nystagmus was regarded as an important diagnostic factor. So, nystagmus examination is very important for the diagnosis of BPPV (Wang et al., 2014).

Some nystagmus can be observed by doctors with naked eyes or Frenzel googles that allow for better visualization of nystagmus at the bedside. However, this diagnostic method was easily affected by the subjective experience of doctors (Slama et al., 2017). Not all pathological nystagmus was visible to the naked eye, since visual fixation suppresses peripheral spontaneous nystagmus. The other method is objective. This method usually uses electronystagmography (ENG) or video nystagmography (VNG) to record eye movements. The ENG method (Costa et al., 1995; Cesarelli et al., 1998) places sensors around the eyelid. Benign positional nystagmus arising from stimulation stimulation of one or more semicircular canals produces horizontal, vertical and torsional eye movements in the plane of that canal. The potential difference measured by the sensors is related to the horizontal and vertical movement of eyes. The velocity and frequency of eye movements can be obtained through potential difference analysis. BPPV also demonstrates a crescendo decrescendo velocity profile, the identification of which could assist with separation of BPV from its mimics. However, this method is vulnerable to electromagnetic interference, in which case the measured information is not accurate enough. The VNG methods generally uses infrared camera to obtain nystagmus video. The frequency and amplitude of nystagmus were obtained by analyzing the motion information of pupil in video (Eggers et al., 2019). Recognizing 3D eye movement trajectory assists in identifying the canal of origin in patients with BPPV.

Under normal test conditions, VNG system includes a series of visual and dynamic function tests (Halmágyi et al., 2001; Newman-Toker et al., 2008). At present, some researchers (Buizza et al., 1978; Van Beuzekom and Van Gisbergen, 2002; Akman et al., 2006) have done some related work on how to use technical means to detect nystagmus. Most of the proposed methods can not fully recognize nystagmus automatically. Some parts or stages of these methods need human intervention, for example, a recognition method of nystagmus proposed by Buizza et al. (1978). In this method, doctors need to calibrate the direction of phase change. Akman et al. proposed a method to detect the period of nystagmus (Akman et al., 2006). The confirmation of the end point of nystagmus still needs to be further improved with this method. Van et al. proposed a nystagmus recognition method with VNG technology (Van Beuzekom and Van Gisbergen, 2002). This method requires researchers to manually confirm the two endpoints of the phase and remove interference factors such as noise from videos.

The research work stated above can be summarized as invasive and non-invasive (Newman et al., 2019). Invasive methods, such as electromagnetic coil method, mainly embed hardware equipment into human eyes, which leads to direct contact between equipment and human eyes. This will cause direct or potential harm to human eye health. The non-invasive detection methods were mainly gaze description methods based on video image processing. These methods detect and locate the pupil based on the contours of the eyes, which were greatly improved in comfort and accuracy.

The Non-invasive inspection methods can be combined with artificial intelligence (AI) methods. At present, AI technology is developing rapidly. Deep Learning has promoted the development of Computer Vision (He et al., 2016; Mane and Mangale, 2018; Kim and Ro, 2019; Cong et al., 2020), Natural Language Processing (Karpathy and Fei-Fei, 2014; Sundermeyer et al., 2015; Young et al., 2018) and other technologies. The development of deep learning technology also provides the possibility for medical intelligent aided diagnosis. For example, CT images of thoracic nodules were analyzed to determine whether there was a tumor in the chest (Anthimopoulos et al., 2016; Setio et al., 2016). Other medical applications include automatic analysis of skin disease images (Rathod et al., 2018; Wu et al., 2019), automatic analysis of fundus disease images (Ting et al., 2017; Sertkaya et al., 2019) and automatic analysis of tumor pathological sections (Tra et al., 2016; Lavanyadevi et al., 2017), etc. A variety of algorithms based on deep learning were integrated into the innovative diagnosis and treatment system (Litjens et al., 2017). For example, Google used neural network to analyze diabetic retinopathy, and its analysis results were similar to those of human experts (Gulshan et al., 2016). In other application fields, deep learning has been applied to motion detection in videos and achieved good recognition results (Saha et al., 2016). Therefore, the recognition of nystagmus can be tried by using the method of deep learning. At present, many scholars have begun to use artificial intelligence methods to identify nystagmus (Zhang et al., 2021; Lu et al., 2022; Wagle et al., 2022). From the experimental results, the deep learning method can be used to detect nystagmus, and the recognition accuracy can be further improved.

This paper mainly focuses on a torsional nystagmus recognition method based on deep learning. With the development of deep learning technology, this paper proposed an automatic recognition method of torsional nystagmus based on deep learning technology to help doctors make rapid diagnosis.

## 2. Materials and methods

### 2.1. Detail of data sources

The data set of this paper comes from Eye, Ear, Nose and Throat (Eye & ENT) Hospital of Fudan University. In the process of data acquisition, the infrared videos were obtained from eye movement recorder with the model of VertiGoggles R ZT-VNG-II, which was provided by Shanghai Zhiting Medical Technology Co., Ltd. Eye movement recorder was used to record and save the patient’s eye movements video. The video format is MP4. The size of video frame is 640×480 and the frame rate is 60fps. The data set include 26,931 nystagmus videos from 1,236 patients. After removing the abnormal and disturbed data, the remaining 24,521 videos were used as the data set. The length of each nystagmus video was not required to be exactly equal. The length of video in the data set was reduced to 6–10 s. The data were from patients with BPPV. The videos were monocular, including left and right eyes. All data were annotated by four ophthalmologists according to the motion characteristics of torsional nystagmus. 80% of the data were used for training and 20% for verification.

The doctors recruited eligible subjects in the otolaryngology clinic or vestibular function examination room. For patients who complained of positional vertigo, bilateral Dix-Hallpike test was performed first, and then bilateral Roll test was performed. Each body position change was rapid, but not exceeded the patient’s tolerance. In case of atypical symptoms such as hearing loss, severe headache, limb sensation or movement disturbance, consciousness disturbance, ataxia, etc., corresponding audiological or imaging examination was carried out first to eliminate other inner ear or central lesions. After judging that the conditions for enrollment were met, the subjects themselves signed the informed consent form and collected their basic information and contact information. The doctor collected nystagmus videos of patients in the whole process of Dix-Hallpike test suspension sitting position and Epley method reduction. The Epley reposition method maintained each position for at least 30 s until the nystagmus disappears. After the restoration, the subjects rested for 15 min, and then performed Dix-Hallpike test again. The negative person indicated that the restoration was successful. If the first reset failed, the doctor performed the reset again and collected the nystagmus video of the second reset.

### 2.2. Network model structure and classification process

Ethical statement. The study was conducted according to the guidelines of the Declaration of Helsinki and approved by Ethics Committee of the Eye, Ear, Nose and Throat Hospital affiliated to Fudan University (approval number: 2020518). Written informed consent was obtained from all enrolled patients.

**Figure 1:**
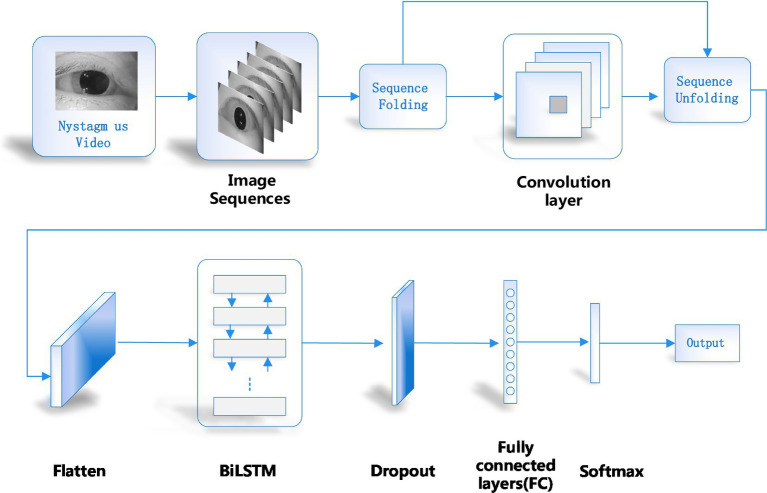
Network model structure of the proposed method.

In order to recognize nystagmus automatically by deep learning, a recognition model as shown in Figure 1 was designed in this paper. Firstly, the nystagmus video was sent to the sequence layer in the model for processing. The output video frame sequence was transmitted to the input of the sequence folding layer. Secondly, the motion characteristics of each frame in the video was extracted independently by convolution operation. Thirdly, the extracted features were restored to the sequence structure after passing through the sequence unfolding layer and flattening layer. At the same time, the output was transformed into vector sequences. Finally, the obtained vector sequences were classified by using Bi-directional Long Short-Term Memory (BiLSTM) layer and output layer. The functions of each part of the network model are introduced as follows.

### 2.3. Converting video into video sequence and sequence folding

Firstly, a single video was processed to obtain the relevant parameters of the video, such as the height, width, number of channels and frames of the video. Then the video was cropped. This paper adopted the longest edge of the cropped video and adjusted its size to obtain a 224 × 224 fixed size. In order to enable the feature extraction network to obtain the features of single frame, a sequence folding layer was constructed to convert sequences into images. The sequence folding layer converts a batch of image sequences into a batch of images. The sequence unfolding layer restores the sequence structure of the input data after the sequence was folded.

### 2.4. Feature extraction

**Figure 2:**
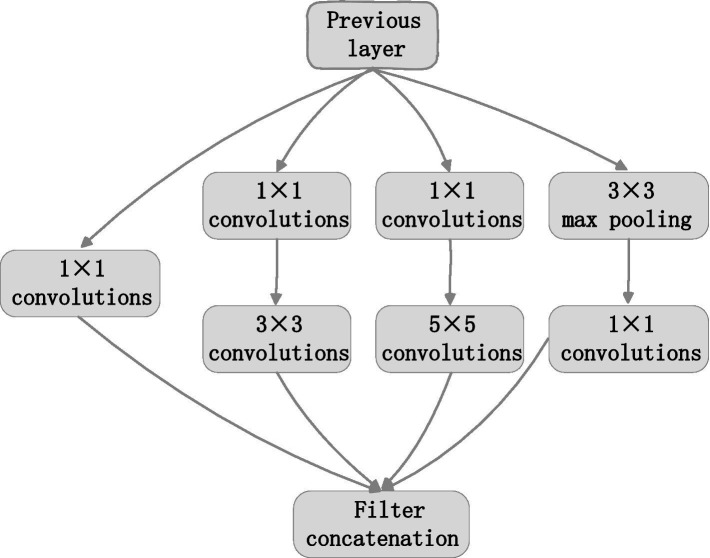
Inception structure.

**Figure 3:**
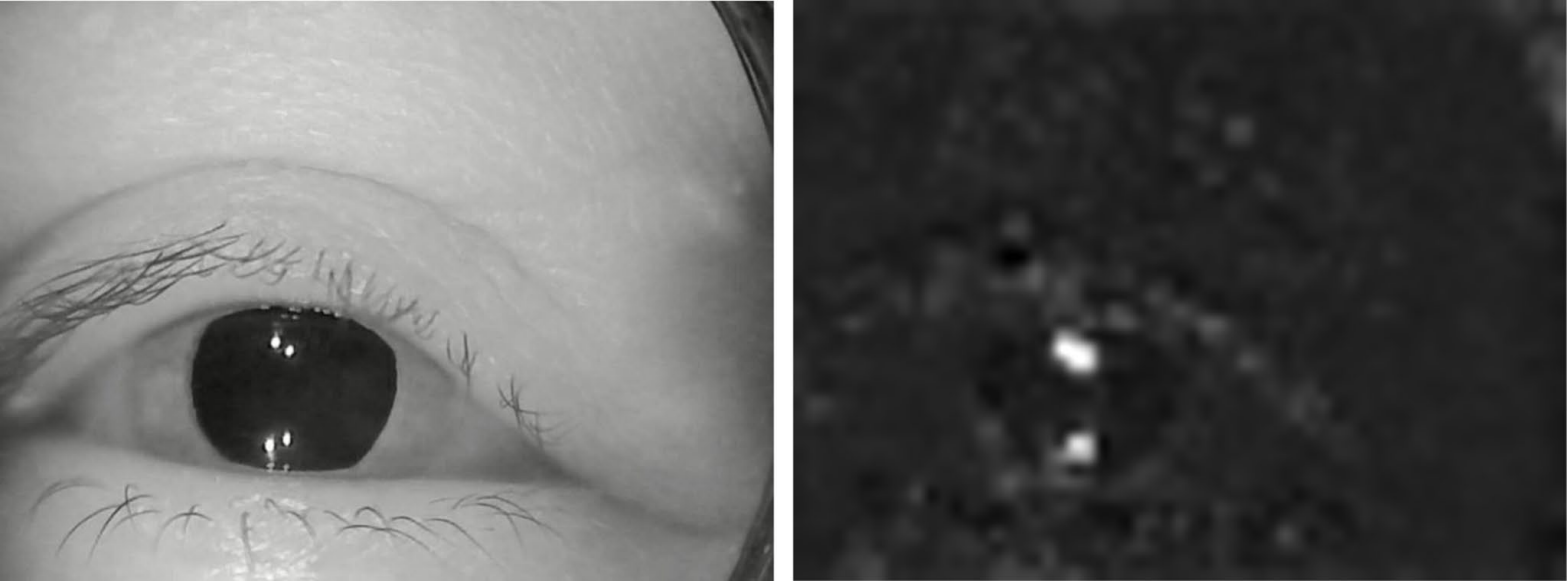
An example of extracted features. **(A)** Original image. **(B)** Extracted features.

Feature extraction was mainly completed by five modules. The first module includes convolution layers and the maximum pooling layer. Convolution layer: the kernel size is 7 × 7; the step of sliding window is 2; the number of output channels is 64. Pooling layer: the window size is 3 × 3; the step of sliding window is 2; the output channel number is 64. The second module has two convolution layers and a maximum pool layer. Convolution layer: the kernel size is 3 × 3; the step of sliding window is 1; the output channel number is 192. Pooling layer: the window size is 3 × 3; the step of sliding window is 2; the output channel number is 192. The third module has two Inception modules in series, followed by a maximum pool layer. Figure 2 illustrates the structure of the Inception module. The Inception module adopts the idea of network in network (NIN). It extracts the local features of the image by using multiple convolution kernels with different scales. Each branch in the Inception module adopts 1 × 1 convolution kernel. It can effectively improve the receptive field of convolution kernel and reduce the dimension to accelerate the network calculation and strengthen the real-time performance. As can be seen from Figure 2, the Inception module has four main components: 1 × 1, 3 × 3, 5 × 5 convolution and 3 × 3 pooling. An example of extracted features in the four components of the inception module was shown in Figure 3. The main purpose of this structure is to extract the multi-scale information through a variety of convolution kernels of different sizes, and then fuse them, so as to have better image representation ability. In practice, using 3 × 3 and 5 × 5 convolution directly will lead to too much calculation. So, 1 × 1 convolution layer should be concatenated in front. The nonlinearity of the network can be increased at the same time.

The numbers of channels output by 4 lines of the first Inception are 64, 128, 32 and 32. The total number of output channels is the accumulation of the four lines, which is 256. The numbers of channels output by 4 lines of the second Inception are 128, 192, 96 and 64 respectively, and the total number of output channels is 480. Pooling layer: the window size is 3 × 3; the step of sliding window is 2; the output channel number is 480.

The fourth module has five Inception blocks in series, followed by a maximum pool layer. The numbers of channels output by 4 lines of the first Inception are 192, 208, 48 and 64 respectively, and the total number of output channels is 512. The numbers of channels output by 4 lines of the second Inception are 160, 224, 64 and 64 respectively, and the total number of output channels is 512. The numbers of channels output by 4 lines of the third Inception are 128, 256, 64 and 64 respectively, and the total number of output channels is 512. The numbers of channels output by 4 lines of the fourth Inception are 112, 288, 64 and 64 respectively, and the total number of output channels is 528. The numbers of channels output by 4 lines of the fifth Inception are 256, 320, 128 and 128 respectively, and the total number of output channels is 832. Pooling layer: the window size is 3 × 3; the step of sliding window is 2; the output channel number is 832.

The fifth module has two Inception blocks in series, followed by a pooling layer. The output channel number of 4 lines are 256, 320, 128 and 128, respectively, in the first Inception, and the total number of output channels is 832. The numbers of channels output by 4 lines of the second Inception are 384, 384, 128 and 128 respectively, and the total number of output channels is 1,024. The pooling layer adopts global average pooling and the convolution layer with height and width of 1 is obtained. The number of output channels is 1,024.

### 2.5. Recovering sequence structure

The sequence structure was deleted by the sequence folding layer. So, the sequence structure should be restored after feature extraction. The recovery task of sequence structure was completed by sequence unfolding layer. The sequence unfolding layer takes the minibatchsize output information of the sequence folding layer as the minibatchsize input information of the sequence unfolding layer. The output of the sequence unfolding layer was reconstructed into vector sequences. The spatial dimension of the tensor was flatted to channel dimension. Flatten layer flattens input spatial dimension into a single channel. This layer retains the observation dimension (*N*) and sequence dimension (*S*) after flattening.

### 2.6. Sequence classification

**Figure 4:**
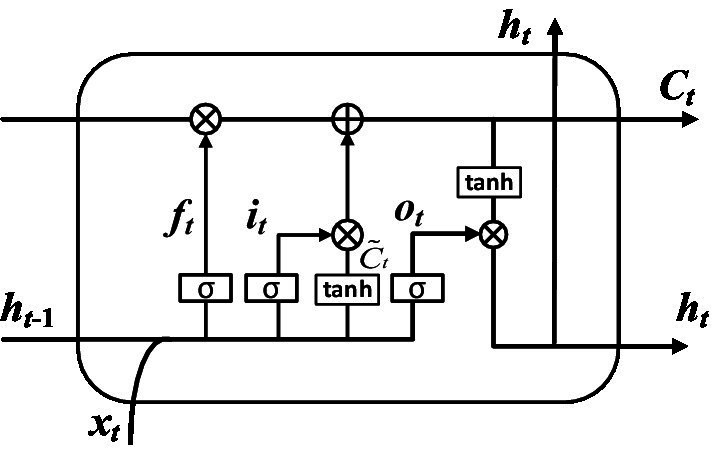
Structure of one-way branching model.

Long Short-Term Memory (LSTM) model can record the relationship between elements in a spatial distance. This memory function can be realized by training LSTM model. But one disadvantage of LSTM model is that the order of memorizing information can only be from front to back. In order to better classify the types of nystagmus, this paper uses BiLSTM to solve this problem. BiLSTM is composed of two LSTMs with opposite directions. Figure 4 shows the structure of the one-way branching model in BiLSTM. In the figure, $x_{t}$, $o_{t}$, $C_{t}$, $f_{t}$, $h_{t}$, ${\tilde{C}}_{t}$ and $i_{t}$ represent input vector, output gate, cell state, forgetting gate, hidden layer state, temporary cell state and memory gate, respectively.

**Figure 5:**
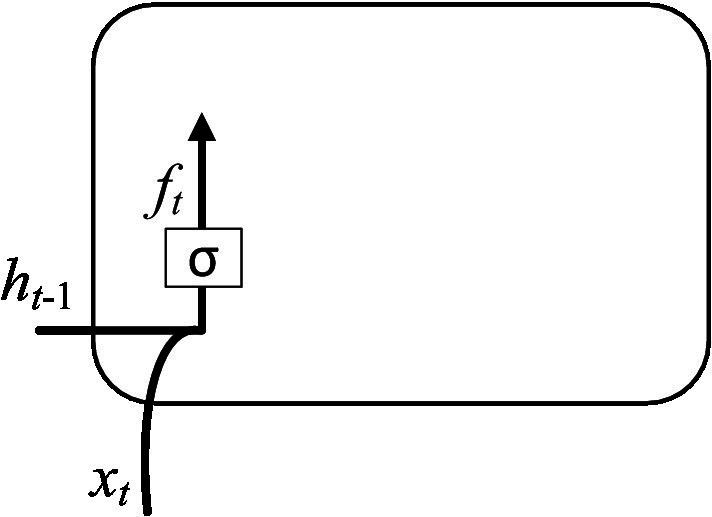
Computation of the forgetting gate.

The classification calculation process was completed by the following steps. Step 1: The discarded information was determined by calculating the forgetting gate. The input is the hidden layer state $h_{t-1}$ at time *t*-1 and the input vector $x_{t}$ at time *t*. The output is the value $f_{t}$ of the forgetting gate at time *t*. As shown in Figure 5.

The input of $h_{t-1}$ and $x_{t}$ were calculated to obtain a forgetting gate output $f_{t}$ through the sigmoid function, and its expression is shown in Equation (1).


(1) $$f_{t}=\sigma \left(w_{f}\cdot \left[h_{t-1},\;x_{t}\right]+b_{f}\right)$$

Where $f_{t}\in \left[0,1\right]$ (0 indicates to discard the information completely, and 1 indicates to retain the information completely); σ indicates the activation function; $w_{f}$ represents a learnable connection vector; $x_{t}$ is input; $b_{f}$ represents the offset value.

**Figure 6:**
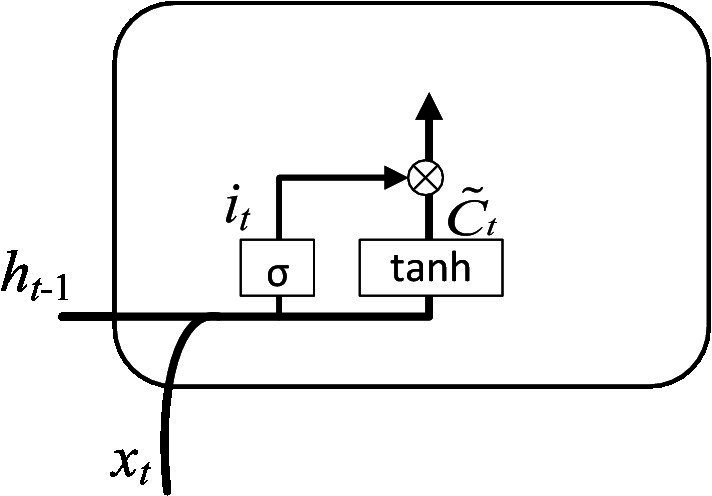
Calculation of the input gate.

Step 2: The retained information was determined by calculating the memory gate. The input is the hidden layer state $h_{t-1}$ at time *t*-1 and the input vector $x_{t}$ at time *t*. The output is the value $i_{t}$ of the memory gate at time *t* and the value ${\tilde{C}}_{t}$ of the temporary cell state at time *t*. As shown in Figure 6. The value of the memory gate was obtained after that the hidden layer state value at time *t*-1 and the input vector at time *t* pass through the sigmoid activation function. The value of the temporary cell state was obtained after the hidden layer state value at time *t*-1 and the input vector at time *t* pass through the tanh activation function. The output values of two activation functions were multiplied to obtain the value of the input gate. The corresponding equation can be written as:


(2) $$i_{t}=\sigma \left(W_{i}\cdot \left[h_{t-1},\;x_{t}\right]+b_{i}\right)$$

(3) $${\tilde{C}}_{t}=\tanh\left(W_{c}\cdot \left[h_{t-1},\;x_{t}\right]+b_{c}\right)$$

Where: *tanh* represents the activation function; $W_{i}$ and $W_{c}$ represent the learnable connection vectors; $b_{i}$ and $b_{c}$ represent the offset values.

**Figure 7:**
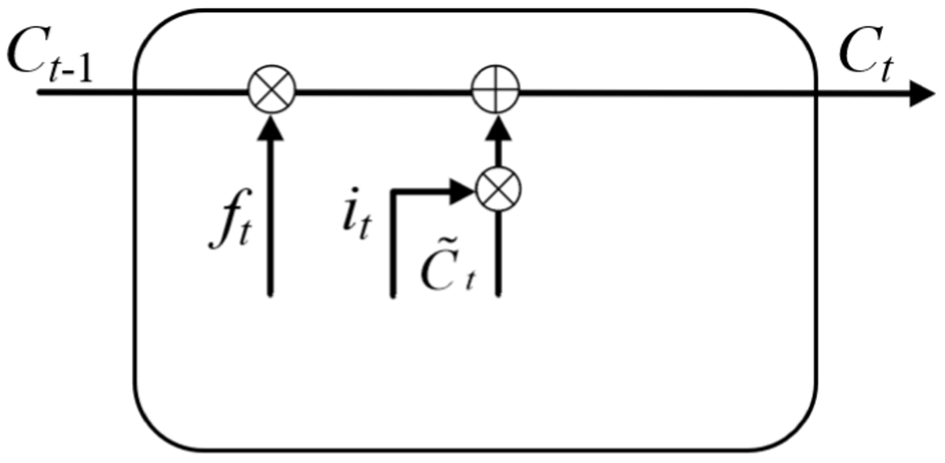
Calculation of the current cell state.

Step 3: The cell state $C_{t}$ was obtained through the joint action of forgetting gate and input gate. The input is the memory gate $i_{t}$ at time *t*, the forgetting gate $f_{t}$ at time *t*, the temporary cell state ${\tilde{C}}_{t}$ at time *t* and the cell state $C_{t-1}$ at time *t*-1. The output is the cell state $C_{t}$ at time *t*. As shown in Figure 7. The corresponding equation can be written as:


(4) $$C_{t}=f_{t}*C_{t-1}+i_{t}*{\tilde{C}}_{t}$$

**Figure 8:**
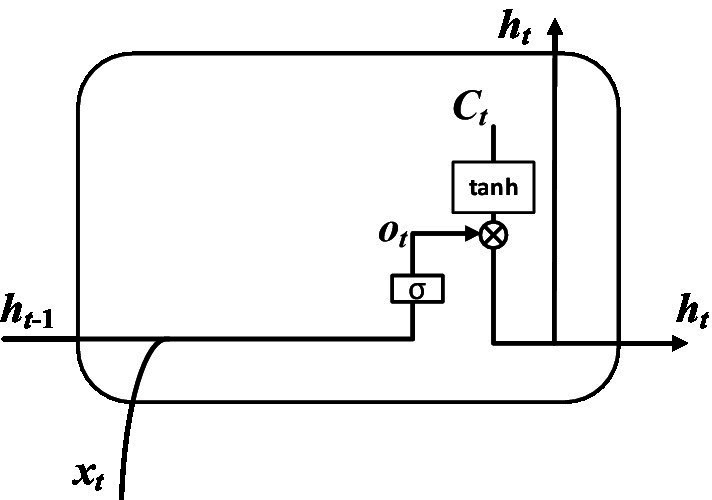
Calculation of hidden layer.

Step 4: The value of the output gate and the value of the hidden layer state were determined by calculation. The input is the hidden layer state $h_{t-1}$ at time *t*-1, the input vector $x_{t}$ at time *t* and the cell state $C_{t}$ at time *t*. The output is the value $o_{t}$ of the output gate at time *t* and the value $h_{t}$ of the hidden layer at time *t*. As shown in Figure 8. The value of output gate was obtained after that the hidden layer state value at time *t*-1 and the input vector at time *t* pass through the sigmoid activation function. The value of hidden layer state was obtained after that the output gate value at time *t* and the cell state at time *t* pass through the tanh activation function. The corresponding expression can be written as:


(5) $$o_{t}=\sigma \left(W_{O}\cdot \left[h_{t-1},\;x_{t}\right]+b_{O}\right)$$

(6) $$h_{t}=o_{t}*\tanh\left(C_{t}\right)$$

$W_{O}$ and $b_{O}$ represent learnable connection vectors and offset values, respectively.

**Figure 9:**
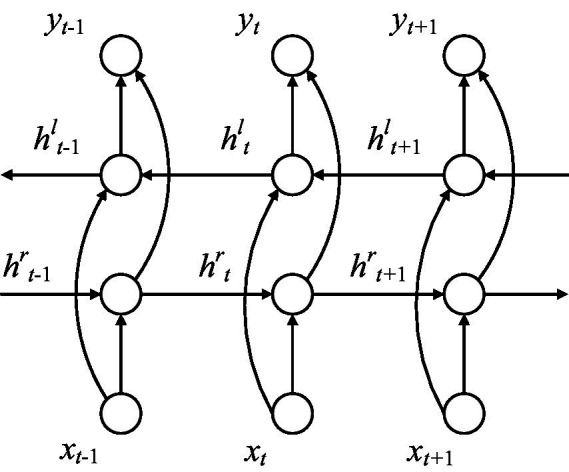
Structure of BiLSTM.

Through the above steps, we can get the corresponding sequence, which is $\;\left\{h_{0},h_{1},\cdots ,h_{n-1}\right\}$. BiLSTM consists of two branches in different directions mentioned above. The parameters on each branch are independent of the other branch. One branch can only fit time-related data from one direction. BiLSTM has two branches in opposite directions so that it can capture patterns that one branch may ignore. The structure can be seen in Figure 9.

If the hidden layer state sequence calculated by one branch of BiLSTM was represented by $h^{r}$, the hidden layer state sequence of the other branche in the opposite direction was represented by $h^{l}$. The final output result is as follow:


(7) $$h_{t}=\alpha h^{r}+\beta h^{l}$$

(8) $$y_{t}=\sigma \left(h_{t}\right)$$

Where α, β are constants and α+β=1. σ is the activation function.

After that the output results pass through the classification layer, the type results of nystagmus recognition can be obtained. The output layer includes dropout layer, full connection layer, softmax layer and classification layer.

## 3. Results

### 3.1. Model training and verification process

**Figure 10:**
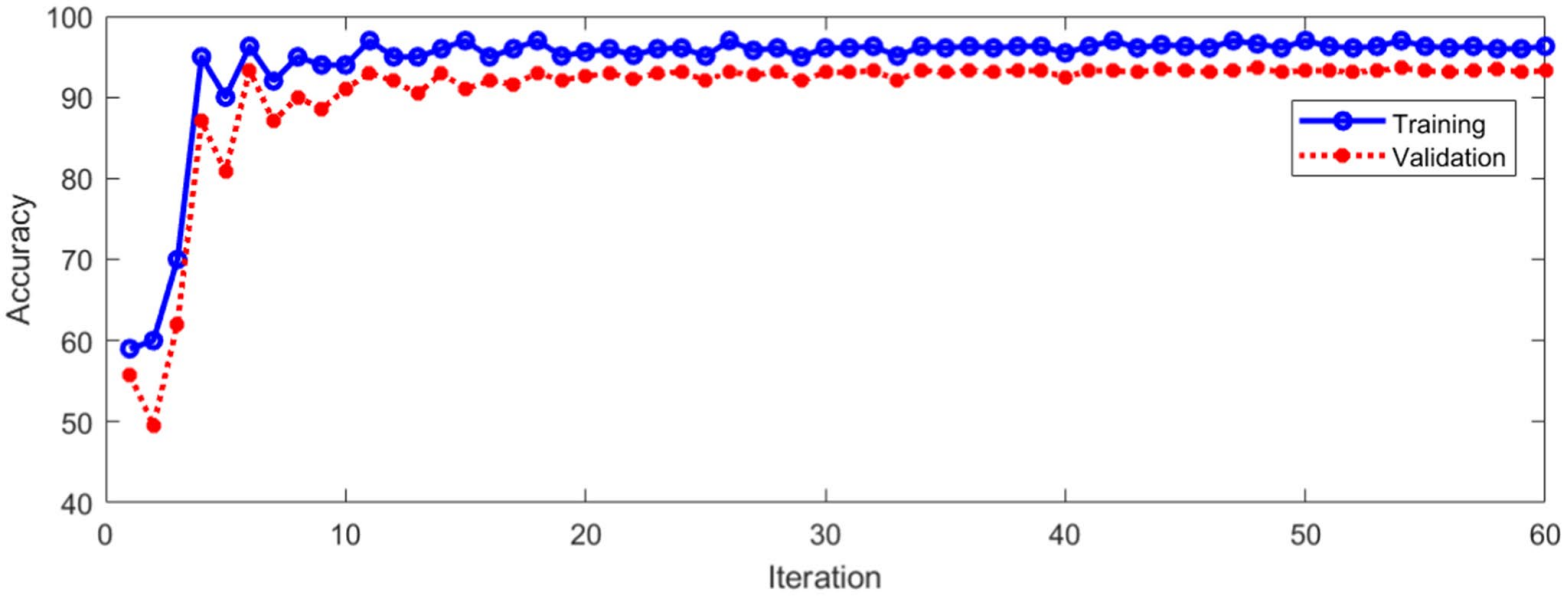
Classification accuracy of training and validation process.

The data set of this paper comes from Eye, Ear, Nose and Throat (Eye & ENT) Hospital of Fudan University. In the process of data acquisition, the infrared videos were obtained from eye movement recorder with the model of VertiGoggles R ZT-VNG-II, which was provided by Shanghai Zhiting Medical Technology Co., Ltd. Eye movement recorder was used to record and save the patient’s eye movements video. The video format is MP4. The size of video frame is 640×480 and the frame rate is 60fps. The data set include 26,931 nystagmus videos from 1,236 patients. After removing the abnormal and disturbed data, the remaining 24,521 videos were used as the data set. All data were annotated by four ophthalmologists according to the motion characteristics of torsional nystagmus. 80% of the data were used for training and 20% for verification. The model training and verification process are shown in Figure 10.

Figure 10 shows that the classification accuracy of the training and verification process tends to be stable with the increase of iterations. The average accuracy after stabilization is shown in Table 1.

**Table 1:** Recognition accuracy in training and verification stage.

Recognition accuracy	Stage
96.1%	Training
92.9%	Validation

**Figure 11:**
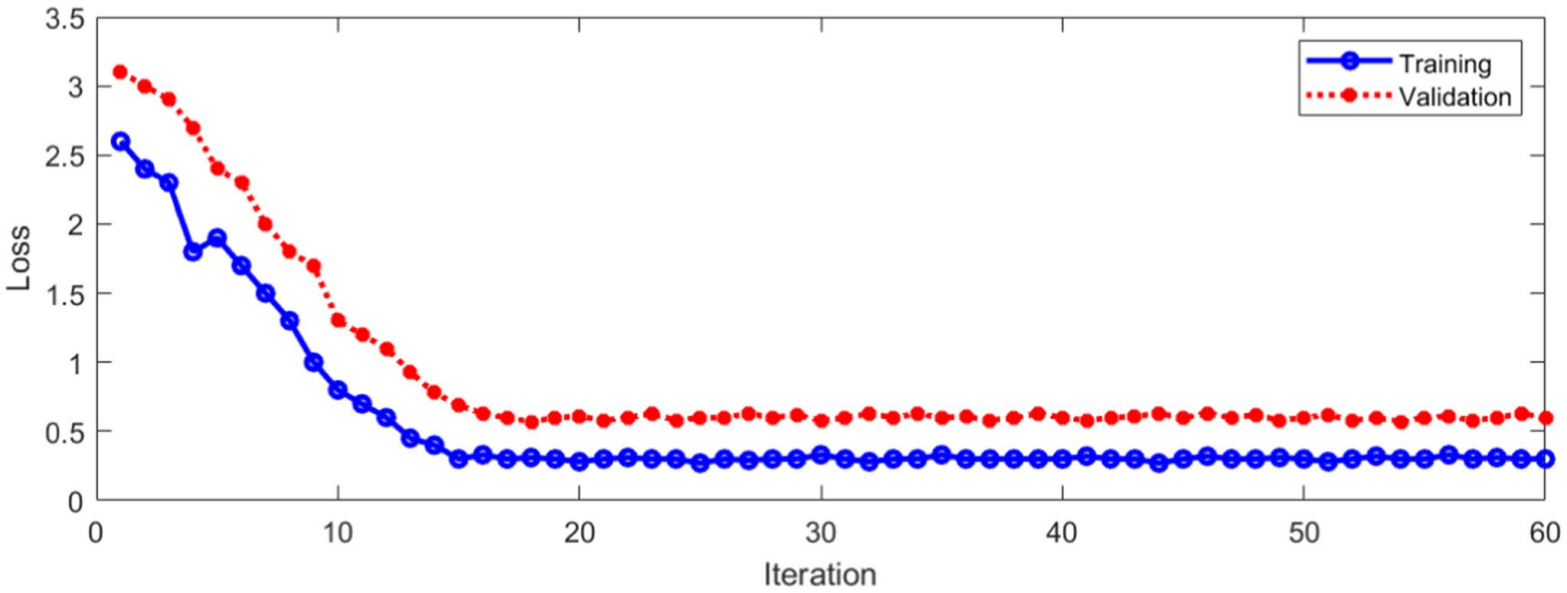
Loss during training and verification.

The loss during training and verification is shown in Figure 11.

**Figure 12:**
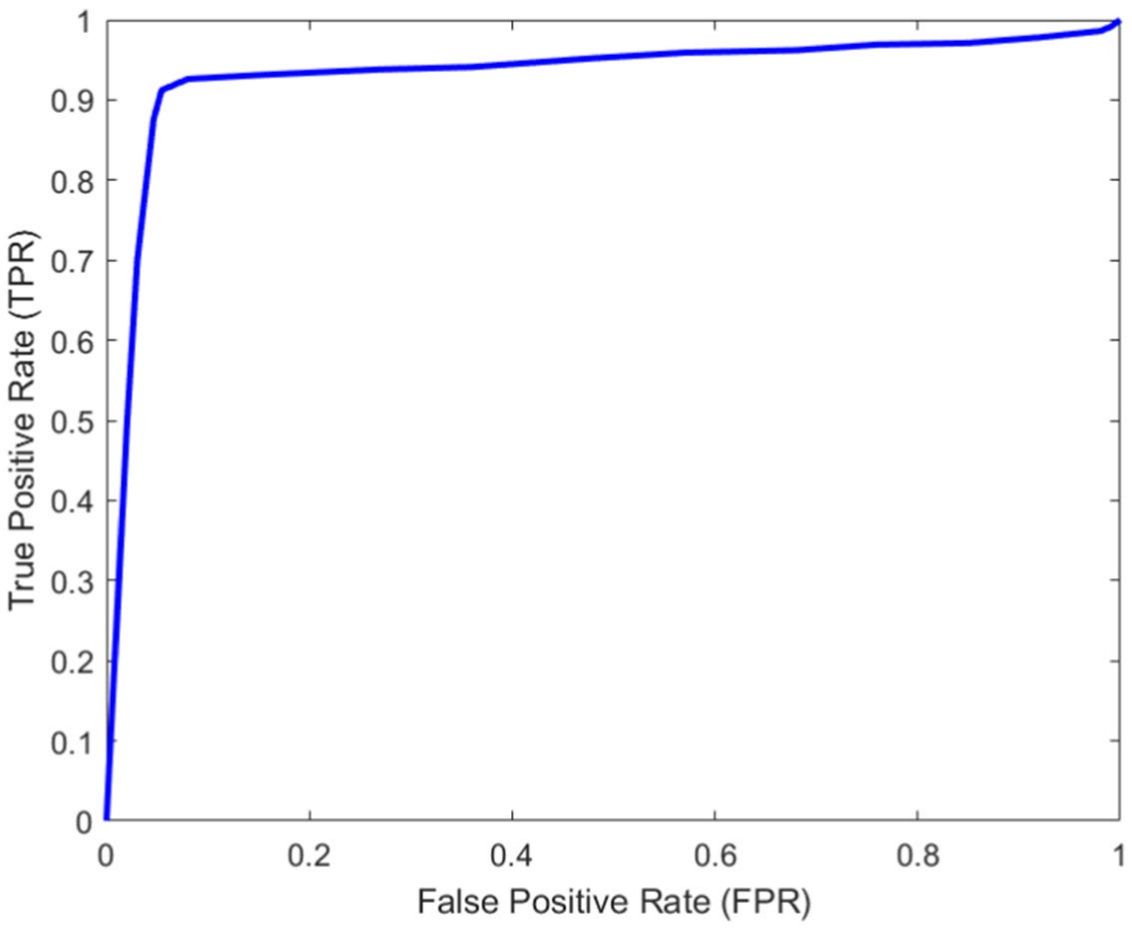
AUC curve.

As can be seen from Figure 11, with the increase of the number of iterations, the loss in the training and verification process has decreased to a stable state. In order to further evaluate the designed method. Figure 12 shows Area Under Curve (AUC).

### 3.2. Results of feature extraction by different methods

**Figure 13:**
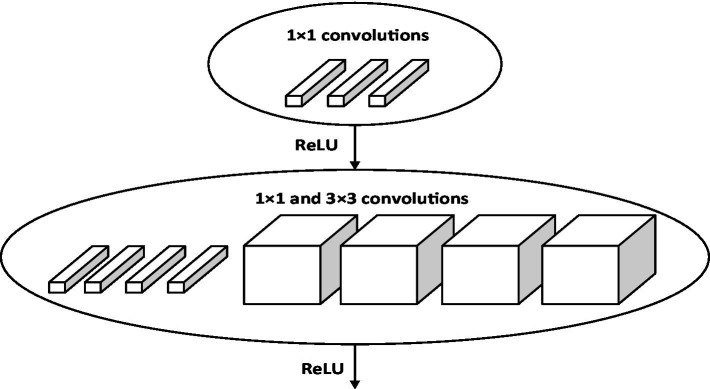
Fire module structure.

In addition, we also study the impact of different methods to extract video frame features on the classification effect. We use eight Fire modules to extract the video frame features, and the structure of other parts remains unchanged. This method is named method 2. The structure of Fire module (Kim and Kim, 2020) is shown in Figure 13.

It can be seen from Figure 12 that the proposed method can identify torsional nystagmus more accurately. In addition, sensitivity ≈0.912 and specificity ≈0.946.

**Figure 14:**
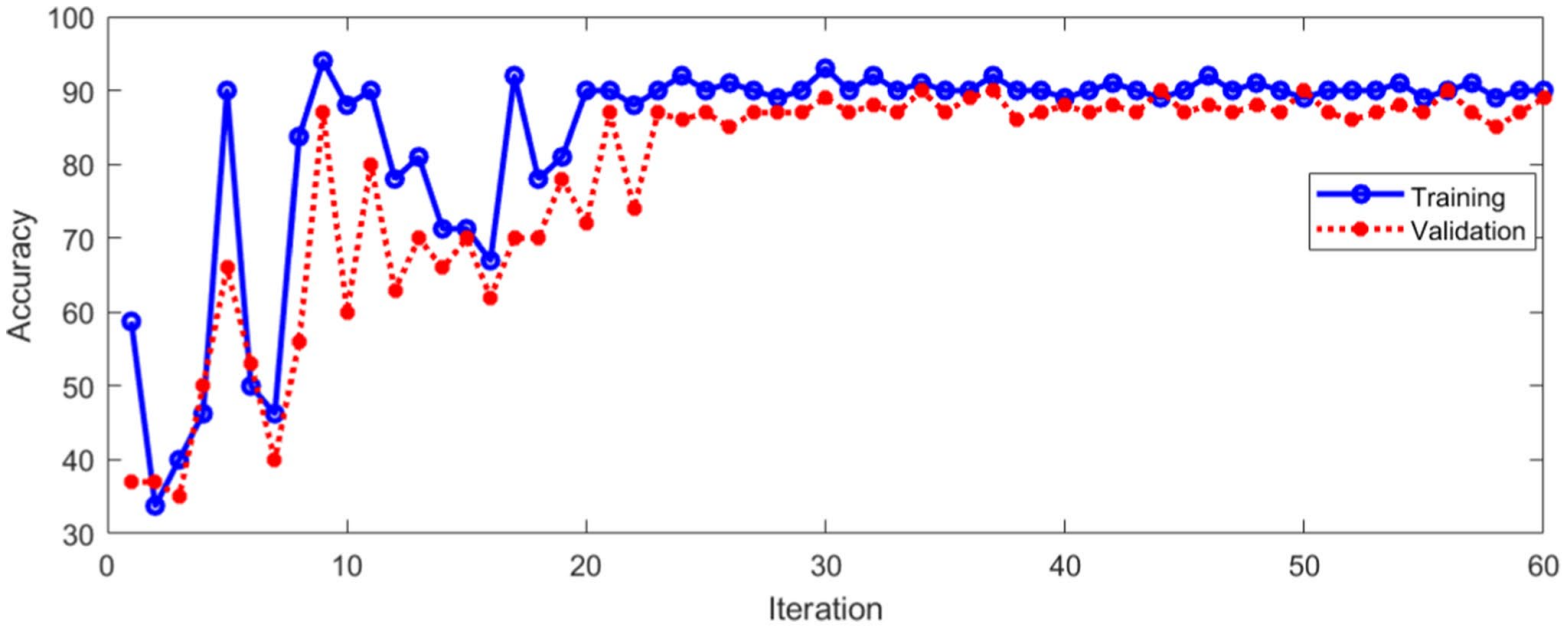
Classification accuracy of method 2.

The same data set was used for training and verification. Figure 14 shows the classification accuracy of training and verification by method 2.

**Figure 15:**
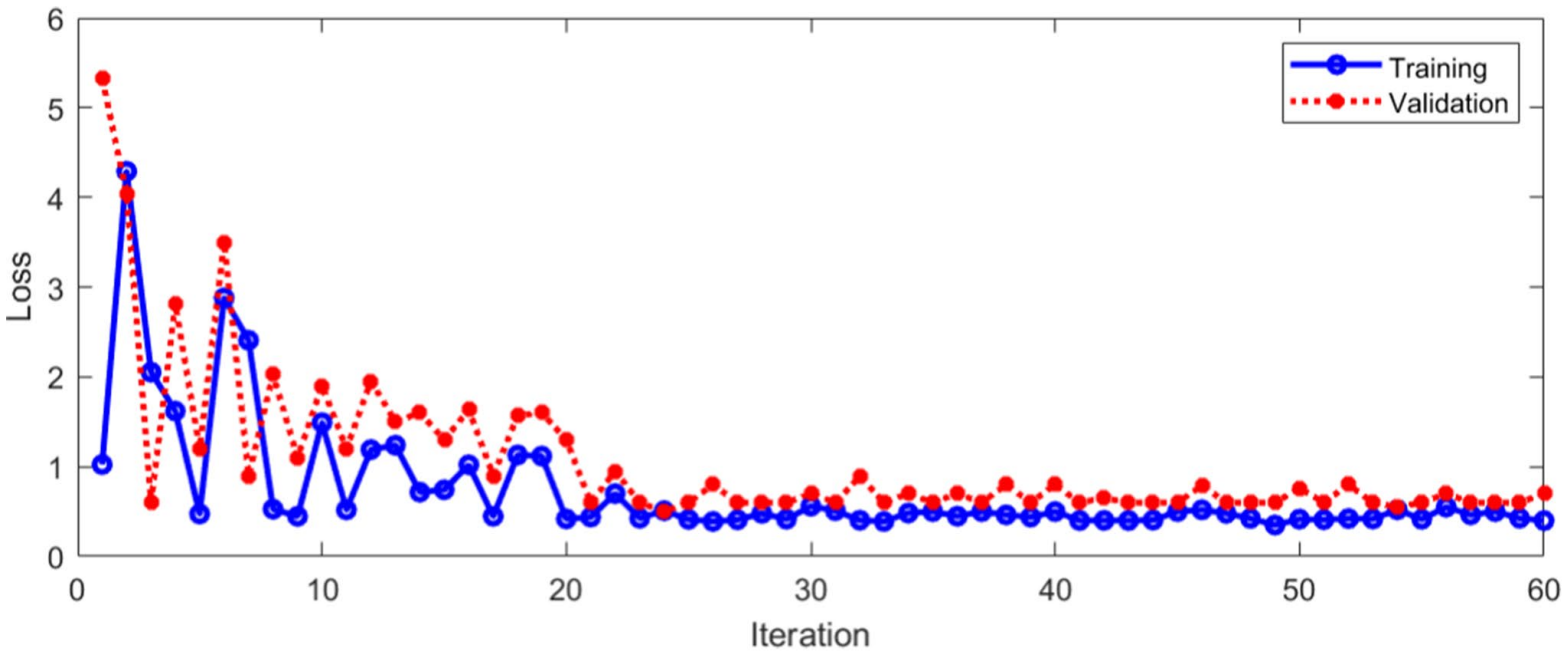
Loss during training and verification with method 2.

As can be seen from Figure 14, the classification accuracy tends to be stable with the increase of iterations, whether in the training process or verification process. Figure 15 shows the Loss during the training and verification process by method 2 using the same data set.

**Figure 16:**
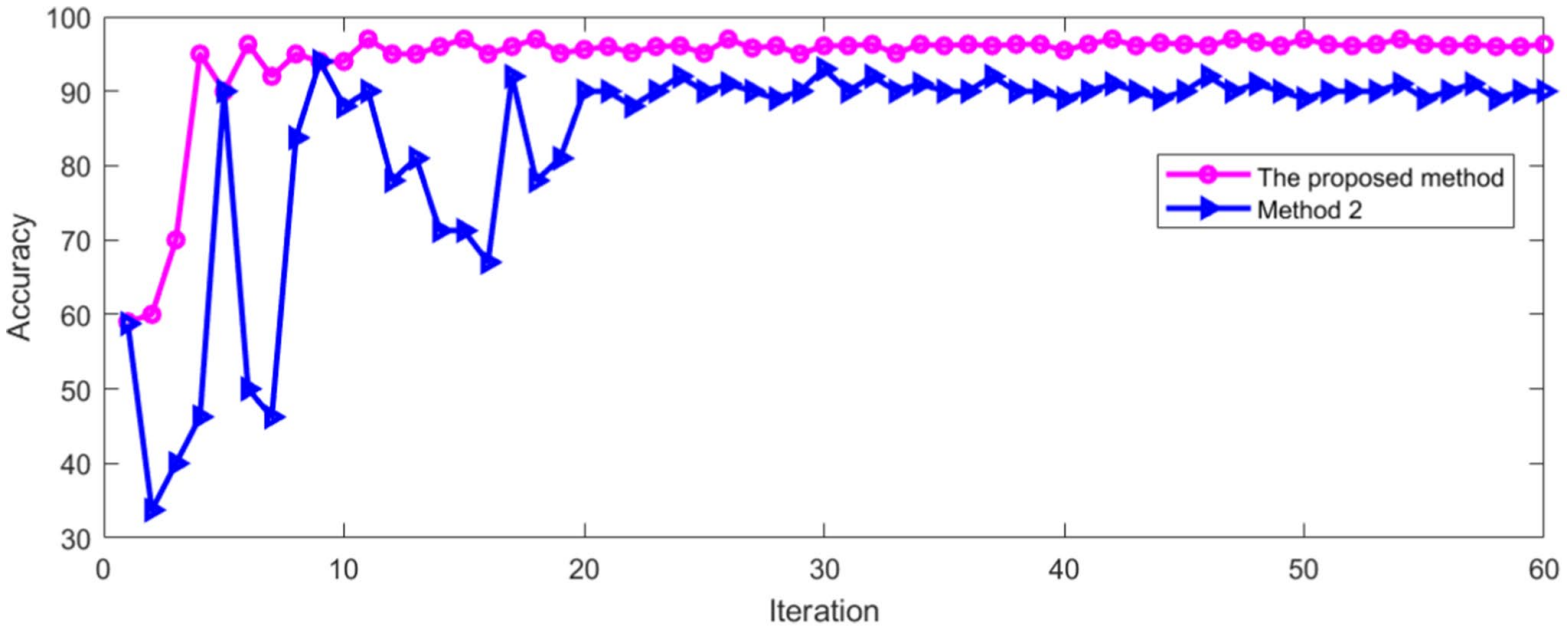
Comparison of accuracy in training set.

As can be seen from Figure 15, with the increase of iterations, the Loss of method 2 decreased to a stable state, whether in the training process or verification process. Method 2 was compared with the method proposed in this paper. The comparison results of classification accuracy in training set are shown in Figure 16.

As can be seen from Figure 16, the recognition accuracy of two methods tends to be stable with the increase of iterations. The average accuracy after stabilization is shown in Table 2.

**Table 2:** Recognition accuracy of two methods in training set.

Recognition accuracy	Method
96.1%	The proposed method
89.9%	Method 2

**Figure 17:**
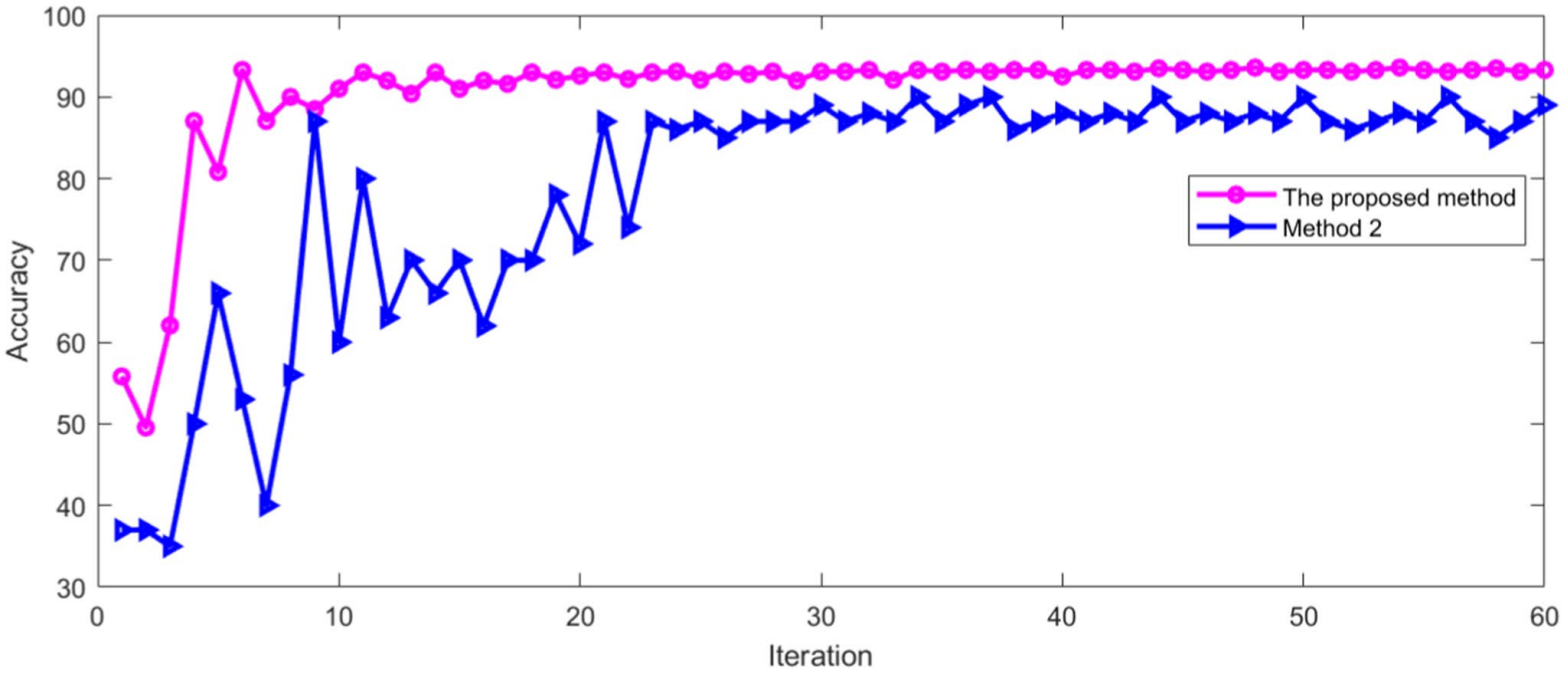
Comparison of accuracy in verification set.

It can be seen from Table 2 that the proposed method has high recognition accuracy in training process. The recognition accuracy of two methods in verification set is shown in Figure 17.

Figure 17 shows that the recognition accuracy of two methods in verification set tends to be stable with the increase of iterations. The average recognition accuracy of two methods after stabilization is shown in Table 3.

**Table 3:** Recognition accuracy of two methods in verification set.

Recognition accuracy	Method
92.9%	The proposed method
87.4%	Method 2

It can be seen from Table 3 that the proposed method has high recognition accuracy in verification set.

## 4. Discussion

**Figure 18:**
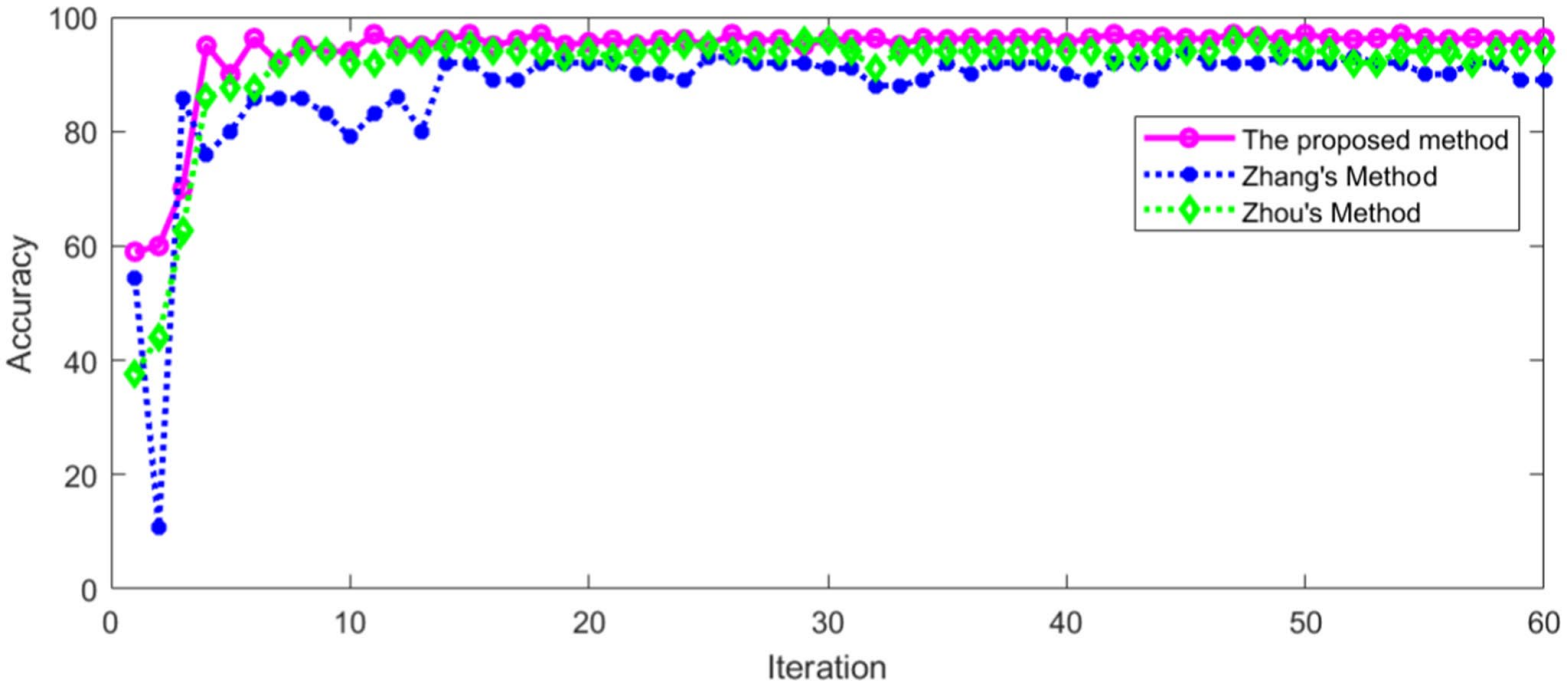
Comparison of accuracy in training set.

The proposed method was compared with Zhang’s method (Zhang et al., 2021) and Zhou’s method (Zhou et al., 2022). The same data set was used for training and verification, respectively. The recognition accuracy of different methods in training set is shown in Figure 18.

As can be seen from Figure 18, the recognition accuracy of all methods in training set tends to be stable with the increase of iterations. The average recognition accuracy of different methods after stabilization is shown in Table 4.

**Table 4:** Recognition accuracy of different methods in training set.

Recognition accuracy	Method
96.1%	The proposed method
91.2%	Zhang’s method
93.9%	Zhou’s method

**Figure 19:**
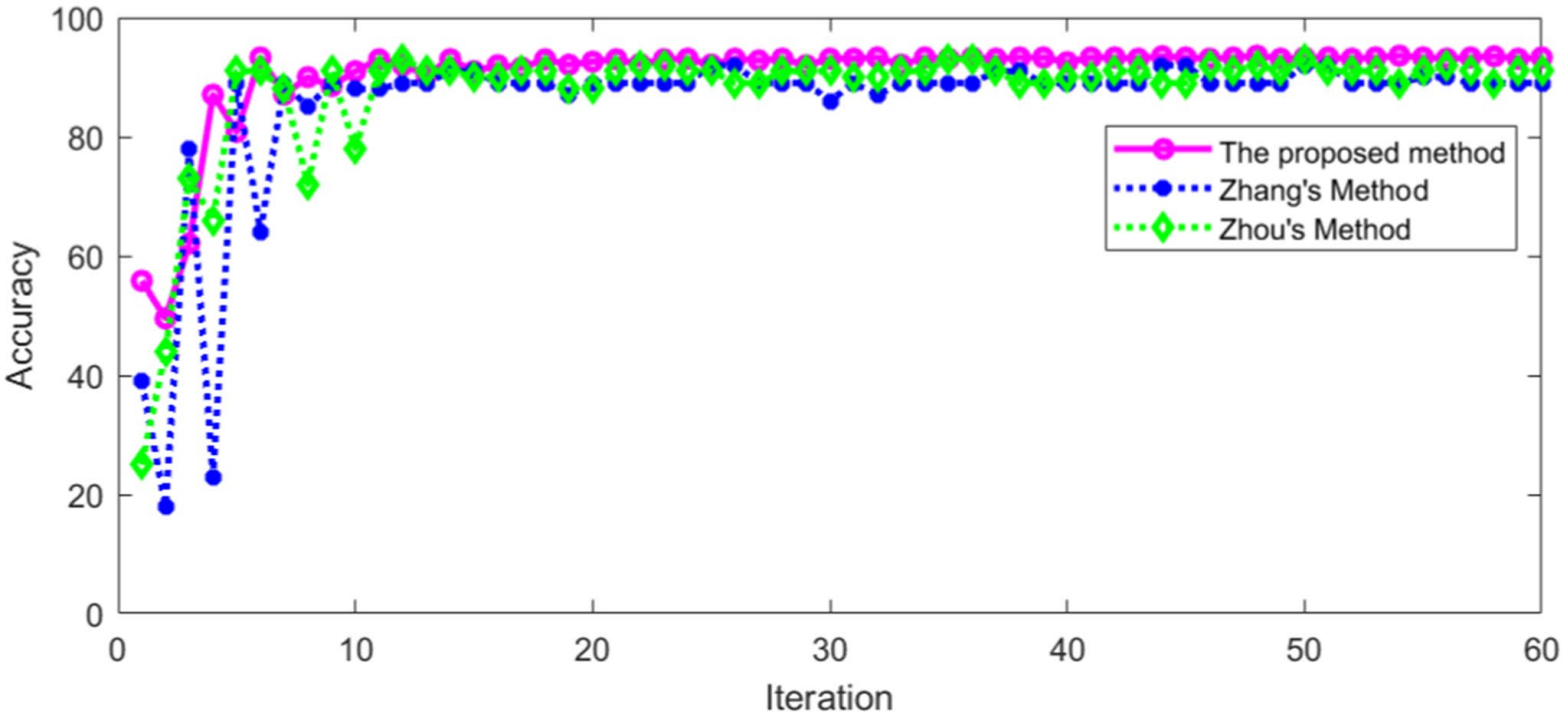
Comparison of accuracy in verification set.

It can be seen from Table 4 that the proposed method has high recognition accuracy in training set. The recognition accuracy of different methods in verification set is shown in Figure 19.

Figure 19 shows that the recognition accuracy of different methods in verification set tends to be stable with the increase of iterations. The average recognition accuracy of different methods after stabilization is shown in Table 5.

**Table 5:** Recognition accuracy of different methods in verification set.

Recognition accuracy	Method
92.9%	The proposed method
89.4%	Zhang’s method
90.7%	Zhou’s method

It can be seen from Table 5 that the proposed method in this paper has high recognition accuracy in verification set. This shows that the proposed method has a good effect in torsional nystagmus recognition. In addition, other statistical comparisons of the variable performance accuracy across models are shown in Table 6.

**Table 6:** Statistical comparisons of the variable performance accuracy.

Method	Precision	Recall
The proposed method	94.3%	91.2%
Zhang’s method	90.1%	87.6%
Zhou’s method	91.9%	88.4%

It can be seen from Table 6 that the proposed method has high precision and recall rate, which indicates that the recognition performance of the algorithm is better than other methods. In addition, we also compared with the method proposed by Wagle et al. (2022), and the recognition accuracy of their method is 82.7%, which is lower than the proposed method.

Based on the real data of a large number of clinical patients, the characteristics and types of torsional nystagmus were intelligently recognized through the deep learning algorithm. The diagnosis of BPPV can be accurately predicted by combining the body position information, so as to realize the intelligent diagnosis and treatment of BPPV, improve the diagnosis efficiency and reduce the pain of patients. It is expected to comprehensively improve the diagnosis and treatment capacity of medical institutions at all levels for typical BPPV patients.

## 5. Conclusion

In this paper, a recognition model of torsional nystagmus was proposed based on deep learning network. From the experimental results, the nystagmus recognition model used convolution neural network to extract the frame features of the video sequence, and classified the obtained vector sequence, which can effectively identify torsional nystagmus. This shows that the recognition of torsional nystagmus can be accomplished by using deep learning network models with different structures. Although these changes in nystagmus are very complex for clinicians, they are indeed extractable features for deep learning. Once these specific nystagmus classification features are obtained, computer-aided clinical screening and classification of typical diseases can widely benefit patients with vertigo disease and help improve the diagnosis efficiency of vertigo disease. Compared with the existing methods, the proposed method further improved the recognition accuracy. In the future, we will label the slow phase velocity (SPV) of the nystagmus, so that we can analyze the performance of the model according to the SPV of the nystagmus. The development of an accurate torsion detection method has implications for correct interpretation of nystagmus overall. BPV is not the only disorder producing torsional nystagmus: stroke, vestibular migraine can present with torsional nystagmus; vestibular neuritis and Menieres disease can also generate horizontal torsional nystagmus. Our present work complements existing methods of 2D nystagmus analysis and could improve the diagnostic capabilities of VNG in multiple vestibular disorders. To automatically pick BPV requires detection of nystagmus in all 3 planes and identification of a paroxysm. This is the next research work to be carried out.

## Data availability statement

The data that support the findings of this study are available from the corresponding author upon reasonable request.

## Ethics statement

The study was conducted according to the guidelines of the Declaration of Helsinki and approved by Ethics Committee of the Eye, Ear, Nose and Throat Hospital affiliated to Fudan University (Approval number: 2020518). Written informed consent was obtained from all enrolled patients.

## Author contributions

HL wrote the main manuscript. ZY prepared the Figures 16, 17. All authors contributed to the article and approved the submitted version.

## Funding

This work was funded by the Shanghai Hospital Development Center, grant number SHDC2020CR3050B.

## Conflict of interest

The authors declare that the research was conducted in the absence of any commercial or financial relationships that could be construed as a potential conflict of interest.

## Publisher’s note

All claims expressed in this article are solely those of the authors and do not necessarily represent those of their affiliated organizations, or those of the publisher, the editors and the reviewers. Any product that may be evaluated in this article, or claim that may be made by its manufacturer, is not guaranteed or endorsed by the publisher.
